# High quality draft genome sequence of *Flavobacterium rivuli* type strain WB 3.3-2^T^ (DSM 21788^T^), a valuable source of polysaccharide decomposing enzymes

**DOI:** 10.1186/s40793-015-0032-y

**Published:** 2015-07-30

**Authors:** Richard L. Hahnke, Erko Stackebrandt, Jan P. Meier-Kolthoff, Brian J. Tindall, Sixing Huang, Manfred Rohde, Alla Lapidus, James Han, Stephan Trong, Matthew Haynes, T.B.K. Reddy, Marcel Huntemann, Amrita Pati, Natalia N. Ivanova, Konstantinos Mavromatis, Victor Markowitz, Tanja Woyke, Markus Göker, Nikos C. Kyrpides, Hans-Peter Klenk

**Affiliations:** Leibniz Institute DSMZ – German Collection of Microorganisms and Cell Cultures, Inhoffenstraße 7B, Braunschweig, Germany; Helmholtz Centre for Infection Research, Inhoffenstraße 7, Braunschweig, Germany; St. Petersburg State University, St. Petersburg, Russia; Algorithmic Biology Lab, St. Petersburg Academic University, St. Petersburg, Russia; DOE Joint Genome Institute, Walnut Creek, California, USA; Biological Data Management and Technology Center, Lawrence Berkeley National Laboratory, Berkeley, CA USA; School of Biology, King Abdulaziz University, Jeddah, Saudi Arabia; School of Biology, Newcastle University, Newcastle upon Tyne, UK

**Keywords:** Carbohydrate active enzyme, Polysaccharide utilization loci, Gram-negative, Non-motile, Aerobic, Hard water rivulet, *Flavobacteriaceae*, Bacteroidetes, GEBA-KMG I, *Myroides*

## Abstract

*Flavobacterium rivuli* Ali et al. 2009 emend. Dong et al. 2013 is one of about 100 species in the genus *Flavobacterium* (family *Flavobacteriacae,* phylum *Bacteroidetes*) with a validly published name, and has been isolated from the spring of a hard water rivulet in Northern Germany. Including all type strains of the genus *Myroides* and *Flavobacterium* into the 16S rRNA gene sequence phylogeny revealed a clustering of members of the genus *Myroides* as a monophyletic group within the genus *Flavobacterium*. Furthermore, *F. rivuli* WB 3.3-2^T^ and its next relatives seem more closely related to the genus *Myroides* than to the type species of the genus *Flavobacterium*, *F. aquatile*. The 4,489,248 bp long genome with its 3,391 protein-coding and 65 RNA genes is part of the ***G****enomic****E****ncyclopedia of****B****acteria and****A****rchaea* project. The genome of *F. rivuli* has almost as many genes encoding carbohydrate active enzymes (151 CAZymes) as genes encoding peptidases (177). Peptidases comprised mostly metallo (M) and serine (S) peptidases. Among CAZymes, 30 glycoside hydrolase families, 10 glycosyl transferase families, 7 carbohydrate binding module families and 7 carbohydrate esterase families were identified. Furthermore, we found four polysaccharide utilization loci (PUL) and one large CAZy rich gene cluster that might enable strain WB 3.3-2^T^ to decompose plant and algae derived polysaccharides. Based on these results we propose *F. rivuli* as an interesting candidate for further physiological studies and the role of *Bacteroidetes* in the decomposition of complex polymers in the environment.

**Electronic supplementary material:**

The online version of this article (doi:10.1186/s40793-015-0032-y) contains supplementary material, which is available to authorized users.

## Introduction

Strain WB 3.3-2^T^ (=DSM 21788^T^ = CIP 109865^T^) is the type strain of *Flavobacterium rivuli* [1, 22]. The genus *Flavobacterium*, the type genus [12, 36] of the family *Flavobacteriaceae* [13], was proposed in the first edition of *Bergey’s Manual of Determinative Bacteriology* in 1923 [10]. *Flavobacteriaceae* have been isolated from soil, freshwater, marine and saline environments [13]. However, members of the *Cytophaga*/*Flavobacteria* group have been found with greater abundances in rivers and oceans [39], which was attributed to their important role in the decomposition of algal-derived organic matter [24, 39, 70]. *F. rivuli* WB 3.3-2^T^ has been isolated from a hardwater rivulet in the Harz Mountains, Germany [17]. Therefore, we selected the freshwater strain WB 3.3-2^T^ as a candidate for comparing its polysaccharide decomposition potential with the one of marine *Flavobacteriaceae*.

Here we present the set of carbohydrate active enzymes, polysaccharide utilization loci and peptidases of strain WB 3.3-2^T^, together with a summary of its present classification, the set of known phenotypic features and a description of the permanent draft genome sequencing and annotation derived from a culture of strain DSM 21788^T^.

## Organism information

### Classification and features

The draft genome of *F. rivuli* DSM 21788^T^ (ARKJ00000000) has one full-length 16S rRNA gene sequence (Q765_20790, 1415 bp) and one partial 16S rRNA gene sequence (Q765_20790, 594 bp) which were both 100 % identical with the sequence from the original species description (AM934661, NR_115084) [1]. BLAST search revealed the presence of a closely related strain CH1-10 (JX971542, 98.4 %) from a mushroom, two closely related (98.5 %) clone sequences from floor dust (FM872607, FM872591) [69], and two clone sequences from human skin (HM274288, HM269957, 98.2 %).

**Fig. 1:**
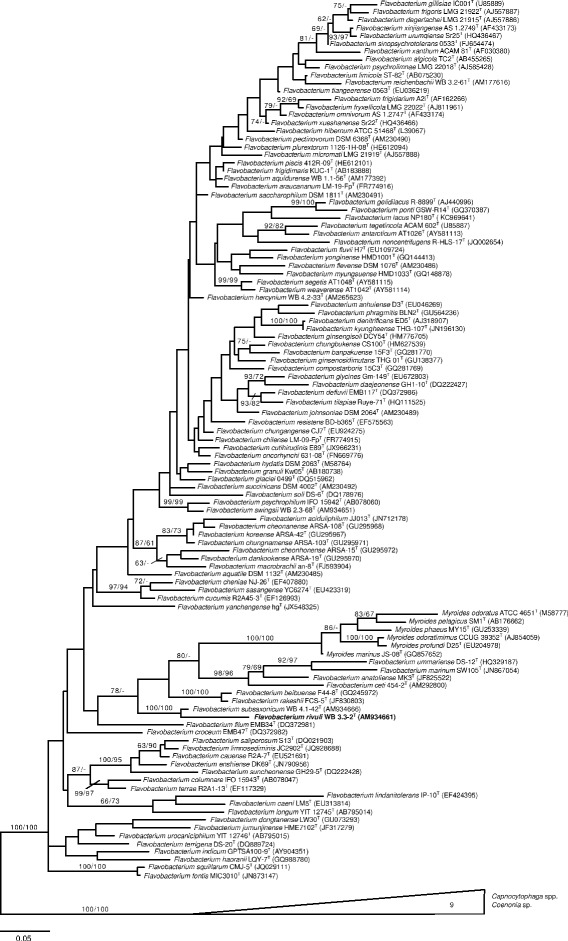
Phylogenetic tree of the genus *Flavobacterium* and its most closely related genus *Capnocytophaga*. The tree was inferred from 1,254 aligned characters of the 16S rRNA gene sequence under the maximum likelihood (ML) criterion as previously described [34]. The sequences were aligned using poa [45] and the resulting alignment restricted to its conserved part using Gblocks [20]. The branches are scaled in terms of the expected number of substitutions per site. Numbers adjacent to the branches are support values from 1,000 ML bootstrap replicates (left) and from 1,000 maximum-parsimony bootstrap replicates (right) if larger than 60 % [34]. Acccession numbers of 16S rRNA gene sequences are listed in Acccession numbers of 16S rRNA gene sequences are listed in Additional file 1: suppl. Table 6

**Fig. 2:**
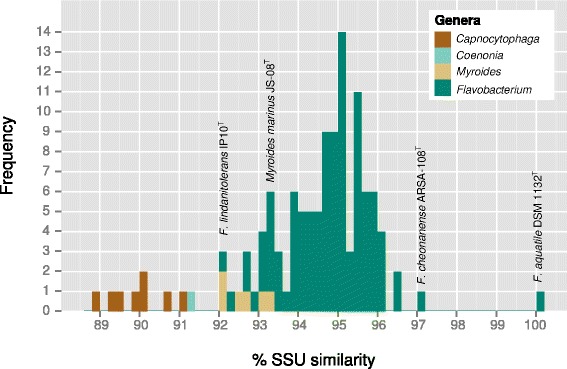
Histogram showing the distribution of pairwise SSU similarities of the type species *Flavobacterium aquatile* with respect to all other 119 strains in the dataset. Except the genus *Myroides*, all genera are clearly segregated from each other. Pairwise SSU similarities were calculated using the recommended approach described in [55]. Bars are colored according to genus affiliation. The figure was visualized using the ggplot package [72] for the R statistical framework [63]. Acccession numbers of 16S rRNA gene sequences are listed in suppl. Table 6

**Fig. 3:**
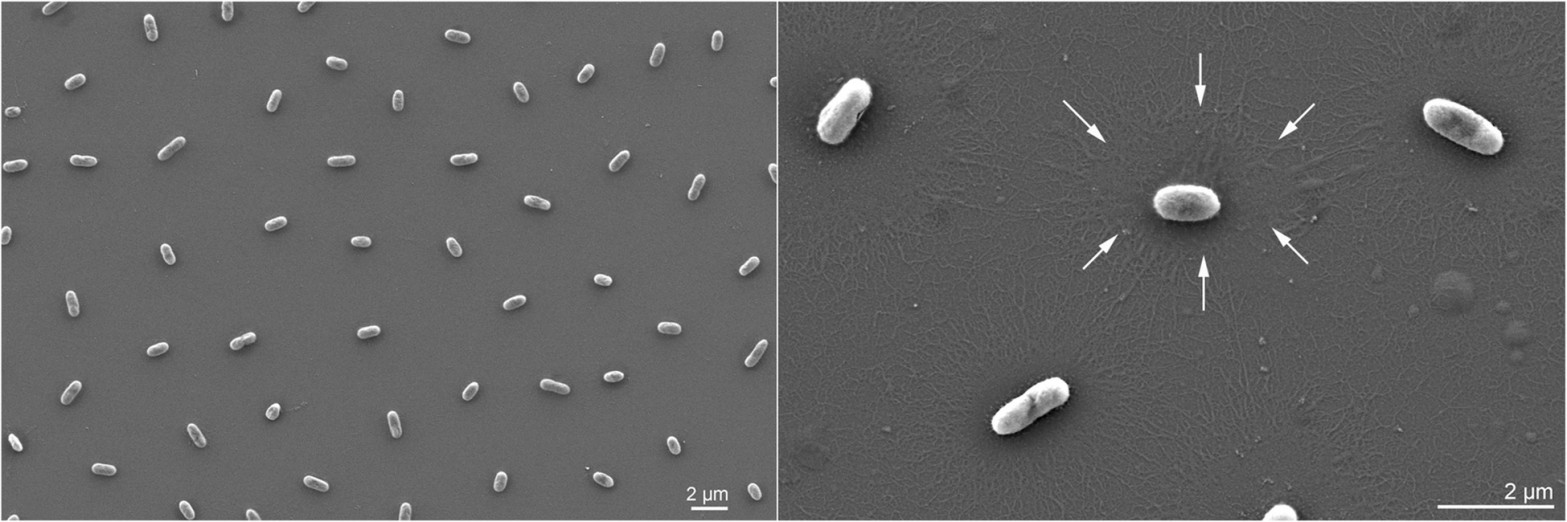
Scanning electron micrograph of *F. rivuli* WB 3.3-2^T^ (DSM 21788^T^) showing expression of extracellular polymeric substances, EPS (arrows)

The next related species was *Flavobacterium**subsaxonensis* WB 4.1-42^T^ [1], whereas other affiliations are poorly supported (Fig. 1). In contrast to the original affiliation with the genus *Flavobacterium*, *F. rivuli* WB 3.3-2^T^ belongs to a group of *Flavobacterium* species which seem more closely related to the genus *Myroides* [71] than to the type species of *Flavobacterium*, *F. aquatile* [10, 15, 29] (Fig. 2). However, the backbone of the 16S rRNA gene phylogenetic tree is essentially unresolved. A summary of the classification and general features of F. rivuli WB 3.3-2T is shown in Table 1. Cells of strain WB 3.3-2^T^ are Gram-negative, aerobic to microaerobic, non-motile (flagella are absent) and non-gliding, catalase- and oxidase-positive 0.4–0.6 × 1.5–2.0 μm rods which produce extracellular polymeric substances (EPS) (Fig. 3). Colonies are pearl-white on R2A and CY agars and yellow on TSA and NA agars. Flexirubin pigments are absent. Sparse growth occurs between 4 and 8 °C and no growth was observed above 29 °C; the growth optimum is between 16 and 24 °C. Growth occurs between pH 6.4 and 7.8 (optimum 7.0) and at NaCl concentrations between 0 and 2 % (w/v) with an optimum at 1 % (w/v). Nitrate reduction is negative. The strain hydrolyses aesculin, cellobiose, glycogen, starch, Tween 40 and Tween 80, but not alginate, caseine, cellulose, chitin, DNA and pectin. The tests for *β*-galactosidase and acid phosphatase are strongly positive. Other physiological properties are available for the API ZYM and API 20NE systems (bioMérieux) and the GN MicroPlate system (Biolog) substrate panels [1]. Maltose and other carbohydrates are assimilated. Properties that can be used for the differentiation from the closely related type strain of *F. subsaxonicum* are, according to the substrates provided by the GN MicroPlate, positive utilization of acetic acid, *α*-d-lactose, trehalose and Tween 40, and lack of utilization of l-alanine, l-fucose, *α*-ketobutyric acid, dl-lactic acid, methyl *ß*-d-glucoside, l-ornithine, l-rhamnose and l-serine.

#### Chemotaxonomic data

Major fatty acids (>5 % of total) are i-C_15:0_, ai-C_15:0_, C_16:0_, C_16:0_ 3-OH, iso-C_17 : 0_ 3-OH and, as main component, summed feature C_16 : 1_ ω7c and/or iso-C_15 : 0_ 2-OH [1]. Although the original publication indicates that “summed feature 3” is present (C_16 : 1_ ω7c and/or iso-C_15 : 0_ 2-OH) and is generally explained as “summed features are groups of two or three fatty acids that cannot be separated by GLC using the MIDI System” this is a misrepresentation of information provided by MIDI Inc as well as a failure to further inspect the final results. Re-examination of the original data held in the DSMZ indicates that a single peak is present with an ECL of 15.819, coinciding with the ECL of C_16 :1_ ω7c in the MIDI Sherlock TSBA40 peak naming table, indicating that C_16:1_ ω7c is present and iso-C_15:0_ 2-OH is absent. While these differences may appear trivial this information can be linked back to the enzymes (their encoding genes) and biosynthetic pathways leading to the synthesis of these two very different fatty acids as has been pointed out previously by [57, 58]. No data are available on respiratory quinone, peptidoglycan, polar lipid, polyamine and whole-cell sugar composition. The DNA G + C content of the type strain was previously determined as 40.4 mol% [1].

### The genera *Flavobacterium* and *Myroides*

Figures 1 and 2 give an overview of the phylogenetic relationships of members of the genus *Flavobacterium* based on the comparison of 16S rRNA gene sequences (see list in Additional file 1: Table S1). In addition members of the genus *Myroides* are included and members of the genus *Capnocytophaga* and *Coenonia* are used as outgroups. Members of the genera *Flavobacterium* and *Myroides* form a monophyletic group, but the division of that monophyletic group to produce a monophyletic group including all members of the genus *Myroides* does not result in members of the genus *Flavobacterium* forming a monophyletic group. In such cases the genus *Flavobacterium* may be divided into several monophyletic groups or the group representing members of the genus *Flavobacterium* and may be described as being paraphyletic. If a genus is to be composed of species that constitute a monophyletic group then the present data suggest at least two alternatives. If one retains the genus *Myroides* as a monophyletic group then the division of the genus *Flavobacterium* into several monophyletic groups may need closer investigation, potentially resulting in the creation of several new genera. Alternatively, the fact that a monophyletic group is recovered that includes members of both the genera *Flavobacterium* and *Myroides* may be indicative of the inclusion of members of both taxa in a single genus, where the genus name *Flavobacterium* Bergey et al. [10] has priority over the genus name *Myroides* Vancanneyt et al. [44, 71]. The type species of the genus *Myroides*, *Myroides odoratus* (Stutzer [68]) Vancanneyt et al. [71] was originally named *F. odoratum* Stutzer [68], i.e. the two names are homotypic synonyms. The lowest 16S rRNA gene sequence pairwise similarity values between the type strain of the type species of the genus *Flavobacterium*, *F. aquatile* and other type strains of species considered to be members of the genus *Flavobacterium* is 92-93 %, close to the 16S rRNA gene sequence pairwise similarity value of 92 % to the type strain of the type species of *Myroides*, *M. odoratum*.

## Genome sequencing information

### Genome project history

*F. rivuli* DSM 21788^T^ was selected for sequencing on the basis of its phylogenetic position [35, 40], and is part of Genomic Encyclopedia of Type Strains, Phase I: the one thousand microbial genomes project [43], a follow-up of the Genomic Encyclopedia of *Bacteria* and *Archaea* pilot project [74], which aims at increasing the sequencing coverage of key reference microbial genomes and to generate a large genomic basis for the discovery of genes encoding novel enzymes [61]. KMG-I is the first of the production phases of the “Genomic Encyclopedia of *Bacteria* and *Archaea*: sequencing a myriad of type strains initiative a [42] and a Genomic Standards Consortium project [27]. The genome project is deposited in the Genomes OnLine Database [59] and the permanent draft genome sequence is deposited in GenBank. Sequencing, finishing and annotation were performed by the DOE Joint Genome Institute (JGI) using state-of-the-art sequencing technology [49]. A summary of the project information is shown in Table 2.

**Table 1:** Classification and general features of *F. rivuli* WB 3.3-2^T^ in accordance with the MIGS recommendations [26], as developed by [25], List of Prokaryotic names with Standing in Nomenclature [23] and the Names for Life database [31]

MIGS ID	Property	Term	Evidence code
	Current classification	Domain *Bacteria*	TAS [73]
		Phylum *Bacteroidetes*	TAS [2, 41]
		Class *Flavobacteriia*	TAS [3, 11]
		Order *Flavobacteriales*	TAS [14, 65]
		Family *Flavobacteriaceae*	TAS [13, 65]
		Genus *Flavobacterium*	TAS [12, 36]
		Species *Flavobacterium rivuli*	TAS [1]
		type strain WB 3.3-2^T^	TAS [1]
	Gram-stain	negative	TAS [1]
	Cell shape	rod-shaped	TAS [1]
	Motility	nonmotile	TAS [1]
	Sporulation	non-spore forming	TAS [13]
	Temperature range	mesophilic (4–29 °C)	TAS [1]
	Optimum temperature	16–24 °C	TAS [1]
	pH range; Optimum	6.4–7.8, 7	TAS [1]
	Carbon source	Carbohydrates, peptides	TAS [1]
MIGS-6	Habitat	fresh water	TAS [1, 17]
MIGS-6.3	Salinity	0–2 %	TAS [1]
MIGS-22	Oxygen requirement	obligate aerobe	TAS [1]
MIGS-15	Biotic relationship	free-living	TAS [1, 17]
MIGS-14	Pathogenicity	not reported	NAS
MIGS-4	Geographic location	Harz Mountains, North Germany	TAS [1, 17]
MIGS-5	Sample collection time	9 June 2005	TAS [1, 17]
MIGS-4.1	Latitude	51.758065	TAS [1, 17]
MIGS-4.2	Longitude	10.11638	TAS [1, 17]
MIGS-4.4	Altitude	273 m	TAS [17]

Evidence codes - IDA, Inferred from Direct Assay (first time in publication), TAS traceable author statement (i.e., a direct report exists in the literature), NAS, non-traceable author statement (i.e., not directly observed for the living, isolated sample, but based on a generally accepted property for the species, or anecdotal evidence). Evidence codes are from the Gene Ontology project [5]

**Table 2:** Genome sequencing project information

MIGS ID	Property	Term
MIGS-31.1	Sequencing quality	Level 2: High-Quality Draft
MIGS-28.1	Libraries method	Illumina Std shotgun library
MIGS-28.2	Reads count	14,972,538 sequencing reads
MIGS-29	Sequencing method	Illumina HiSeq 2000,
MIGS-31.2	Fold coverage	124.1x
MIGS-30	Assembly method	Velvet v. 1.1.04; ALLPATHS v. r41043
MIGS-32	Gene calling method	Prodigal, GenePRIMP, IMG-ER
	NCBI project ID	182404
	Genbank ID	ARKJ00000000
	Genbank Date of Release	16-SEP-2013
	IMG object ID	2519103183
	GOLD ID	Gi11501
MIGS-13	Source Material Identifier	DSM 21788
	Project relevance	Tree of Life, GEBA-KMG

### Growth Conditions and genomic DNA preparation

A culture of DSM 21788^T^ was grown aerobically in DSMZ medium 830 [4] at 20 °C. Genomic DNA was isolated using Jetflex Genomic DNA Purification Kit (GENOMED 600100) following the standard protocol provided by the manufacturer but modified by an incubation time of 60 min. the incubation on ice overnight on a shaker, the use of additional 50 μl proteinase K, and the addition of 200 μl protein precipitation buffer. DNA is available from DSMZ through the DNA Bank Network [32].

### Genome sequencing and assembly

The draft genome of DSM 21788^T^ was generated using the Illumina technology [9]. An Illumina Std. shotgun library was constructed and sequenced using the Illumina HiSeq 2000 platform which generated 14,972,538 reads totaling 2,245.9 Mbp (Table 3). All general aspects of library construction and sequencing performed at the JGI can be found at [21]. All raw sequence data were passed through DUK, a filtering program developed at JGI, which removes known Illumina sequencing and library preparation artifacts (Mingkun L, Copeland A, Han J, DUK. Unpublished). Following steps were performed for assembly: (1) filtered reads were assembled using Velvet [77], (2) 1–3 Kbp simulated paired end reads were created from Velvet contigs using wgsim [46], (3) Sequence reads were assembled with simulated read pairs using Allpaths–LG [33]. Parameters for assembly steps were: 1) Velvet (velveth: 63 –shortPaired and velvetg: –very clean yes –export- Filtered yes –min contig lgth 500 –scaffolding no –cov cutoff 10) 2) wgsim (–e 0 –1 100 –2 100 –r 0 –R 0 –X 0) 3) Allpaths–LG (PrepareAllpathsInputs: PHRED 64 = 1 PLOIDY = 1 FRAG COVERAGE = 125 JUMP COVERAGE = 25 LONG JUMP COV = 50, RunAllpathsLG: THREADS = 8 RUN = std shredpairs TARGETS = standard VAPI WARN ONLY = True OVERWRITE = True). The final draft assembly contained 26 contigs in 23 scaffolds, with three contigs shorter than the threshold used to generate Table 3. The total size of the genome is 4.5 Mbp and the final assembly is based on 560.1 Mbp of data, which provides a 124.1x average coverage of the genome.

**Table 3:** Genome statistics

Attribute	Number	% of Total
DNA, total number of bases	4489248	100.0
DNA coding number of bases	3981399	88.7
DNA G + C number of bases	1777758	39.6
DNA scaffolds	23	100.0
Genes total number	4056	100.0
Protein coding genes	3991	98.4
RNA genes	65	1.6
rRNA genes	8	0.2
5S rRNA	5	0.1
16S rRNA	1	<0.1
23S rRNA	2	0.1
tRNA genes	48	1.2
Other RNA genes	9	0.2
Protein coding genes with function prediction	2842	70.1
without function prediction	1149	28.3
Protein coding genes with COGs	2570	63.4
Protein coding genes with Pfam	2924	72.1
Protein coding genes coding signal peptides	654	16.1
Protein coding genes coding transmembrane proteins	906	22.3
CRISPR repeats	0	

### Genome annotation

Genes were identified using Prodigal [37] as part of the DOE-JGI genome annotation pipeline [49], followed by manual curation using the JGI GenePRIMP pipeline [60]. The predicted CDSs were translated and used to search the National Center for Biotechnology Information (NCBI) non-redundant database, UniProt, TIGR-Fam, Pfam, PRIAM, KEGG, COG, and InterPro database. These data sources were combined to assert a product description for each predicted protein. Additional gene prediction analysis and functional annotation was performed within the Integrated Microbial Genomes-Expert Review (IMG-ER) platform [48].

## Genome properties

The assembly of the draft genome sequence consists of 23 scaffolds amounting to 4,489,248 bp. The G + C content is 39.6 % (Table 3) which is similar to the G + C content determined by Ali et al. [1] and is within the acceptable range for a microbial species [56]. Of the 4,056 genes predicted, 3,991 were protein-coding genes, and 65 RNAs. The majority of the protein-coding genes (70.1 %) were assigned a putative function while the remaining ones were annotated as hypothetical proteins. The distribution of genes into COGs functional categories is presented in Table 4.

**Table 4:** Number of genes associated with the general COG functional categories

Code	Value	% age	Description
J	152	5.4	Translation, ribosomal structure and biogenesis
A	1	0.1	RNA processing and modification
K	209	7.4	Transcription
L	138	4.9	Replication, recombination and repair
B	1	0.1	Chromatin structure and dynamics
D	21	0.8	Cell cycle control, cell division, chromosome partitioning
Y	0	0.0	Nuclear structure
V	49	1.7	Defense mechanisms
T	177	6.3	Signal transduction mechanisms
M	237	8.4	Cell wall/membrane/envelope biogenesis
N	10	0.4	Cell motility
Z	0	0.0	Cytoskeleton
W	0	0.0	Extracellular structures
U	50	1.8	Intracellular trafficking, secretion, and vesicular transport
O	111	3.9	Posttranslational modification, protein turnover, chaperones
C	152	5.4	Energy production and conversion
G	201	7.2	Carbohydrate transport and metabolism
E	199	7.1	Amino acid transport and metabolism
F	67	2.4	Nucleotide transport and metabolism
H	124	4.4	Coenzyme transport and metabolism
I	109	3.9	Lipid transport and metabolism
P	133	4.7	Inorganic ion transport and metabolism
Q	48	1.7	Secondary metabolites biosynthesis, transport and catabolism
R	360	12.8	General function prediction only
S	261	9.3	Function unknown
-	1486	36.6	Not in COGs

## Insights from the genome sequence

### Comparative genomics

Here we present a brief comparative genomics analysis of *F. rivuli* DSM 21788^T^ with a selection of its closest phylogenetic neighbour (according to Fig. 1), *F. subsaxonicum* [1] (NZ_AUGP00000000), other potentially closely related species such as *F. filum* [66] (NZ_AUDM00000000) and *F. beibuense* [30] (NZ_JRLV00000000), as well as the genome of the type species of the genus *Flavobacterium*, *F. aquatile* [10, 15, 29] (NZ_JRHH00000000). The genomes of these five sequenced *Flavobacterium* type strains differ significantly in their size: *F. rivuli* 4.49 Mbp (see above), *F. beibuense* 3.8 Mbp, *F. filum* 3.19 Mbp, *F. subsaxonicum* 4.63 Mbp and *F. aquatile* 3.49 Mbp. Since these genome sequences have not been sequenced completely yet, the final values might change slightly in future analyses based on complete genome sequences.

An estimate of the overall similarity between *F. rivuli* and the other strains in this data set was generated with the Genome-to-Genome Distance Calculator (2.0) [6, 7, 53]. It calculates intergenomic distances by comparing two respective genomes to obtain HSPs (high-scoring segment pairs) and, afterwards, infers distances via a set of formulas (1, HSP length/total length; 2, identities/HSP length; 3, identities/total length). The GGDC also reports model-based DDH estimates (digital DDH or dDDH) along with their confidence intervals [53]. Since formula 2 is robust against the use of incomplete genome sequences (see above), it is especially suited for this type of analysis.

The result of this comparison is shown in Table 5 and yields dDDH of below 22 % throughout, which underlines the expected status of distinct species, as inferred from the 16S rRNA sequence similarities. Consequently, with 21.3 % dDDH *F. subsaxonicum* has the highest similarity to *F. rivuli*, whereas *F. aquatile* has the lowest similarity of 18.4 % dDDH. The comparison of *F. rivuli* with *F. aquatile* and *F. filum* reached the lowest value (2 %) regarding the average genome length covered with HSPs. This value was slightly increased (7 %) between *F. rivuli* and *F. beibuense* and clearly higher (31 %) with respect to *F. subsaxonicum*, the closest related species according to Fig. 1. The identity within the HSPs was 77 % on average, whereas the identity over the whole genome was 24 % regarding the comparison of *F. rivuli* with *F. subsaxonicum*, and, was even below 10 % regarding the remaining comparisons (Table 5).

**Table 5:** Pairwise comparison of *F. rivuli* with *F. filum*, *F. subsaxonicum*, *F. beibuense* and *F. aquatile* using the GGDC (Genome-to-Genome Distance Calculator). Digital DDH (dDDH) and the respective confidence intervals (C.I.) are specified for GGDC’s recommended formula 2

*F. rivuli* versus	% dDDH	% C.I. dDDH	HSP length/total length [%]	Identities/HSP length [%]	Identities/total length [%]
*F. aquatile*	18.4	2.5	2	76	1
*F. beibuense*	18.7	2.6	7	76	6
*F. filum*	19.0	2.5	2	77	1
*F. subsaxonicum*	21.3	2.9	31	79	24

### Gliding motility

The gliding motility machinery among *Bacteroidetes* is composed of adhesion-like proteins, an ATP-binding cassette transporter, the PorS secretion system, and additional proteins, as described by McBride and Zhu [51]. In the genome of *F. rivuli* all genes necessary for gliding motility were identified (Table 6). However, adhesin-like proteins comparable to the ones of *F. johnsoniae* UW101 were not found, and gliding motility of *F. rivuli* was not observed previously [1].

**Table 6:** Gliding motility-related genes in strain DSM 21788^T^ compared to genes in *Flavobacterium* strains studied by McBride and Zhu [51]

		*F. rivuli* DSM 21788^T^	*F. psychrophilum* JIP02/86^T^	*F. johnsoniae* ATCC 17061^T^
Locus tag prefix		F565_ RS01	FP	Fjoh_
Gliding motility		–	+	+
Adhesin-like				
	remA	–	1959	0808
	remB	–	2117	1657
	sprB	–	0016	0979
ATP-binding cassette transporter				
	gldA	05270	0252	1516
	gldF	00760	1089	2722
	gldG	00765	1090	2721
Additional protein required for gliding				
	gldB^a^	13390	2069	1793
	gldC	13385	2068	1794
	gldD^a^	18865	1663	1540
	gldE	18860	1358	1539
	gldH^a^	10515	0024	0890
	gldJ^a^	11845	1389	1557
Peptidoprolyl isomerase (*Flavobacteriia*, protein folding)				
	gldI	08180	1892	2369
PorS secretion system (secretion of RemA/RemB and SprA/SprB)				
	gldK^a^	18605	1973	1853
	gldL^a^	18600	1972	1854
	gldM^a^	18595	1971	1855
	gldN^a^	18590	1970	1856
	sprA^a^	06065	2121	1653
	sprE^a^	19150	2467	1051
	sprT^a^	05475	0326	1466

^a^essential gliding motility genes after McBride and Zhu [51]

### Peptidases

The MEROPS [64] annotation was carried out by searching the sequences against MEROPS 9.10 (access date: 2014.10.16, version: pepunit.lib). *F. rivuli* processes 177 peptidases the majority of which were 59 metallo (M) and 89 serine (S) peptidases (Table 7 and Additional file 1: Table S2). Furthermore, the *F. rivuli* genome contained 22 I39, two I87 and one I71 simple peptidase inhibitors (Table 7 and Additional file 1: Table S3).

**Table 7:** Peptidases and simple peptidase inhibitors in the genome of strain DSM 21788^T^

Peptidase family	M01	M03	M12	M13	M14	M16	M19	M20	M23
Counts	6	2	2	2	8	3	1	5	8
Peptidase family	M24	M28	M38	M41	M42	M43	M48	M50	M61
Counts	2	2	6	1	1	1	1	1	1
Peptidase family	M75	M79	M90	M96					
Counts	2	1	1	2					
Peptidase family	S01	S08	S09	S11	S12	S14	S16	S24	S26
Counts	2	5	31	1	6	3	3	5	1
Peptidase family	S33	S41	S46	S49	S51	S54	S66		
Counts	16	6	3	1	2	3	2		
Peptidase family	C01	C25	C26	C40	C44	C56	C82		
Counts	1	1	8	3	4	4	1		
Peptidase family	N11		T02		U32	U73		A08	A28
Counts	1		1		2	1		1	1
Inhibitor family	I39	I71	I87						
Counts	22	1	2						

### Carbohydrate active enzymes

The CAZyme annotation was a combination of RAPSearch2 search [75, 78] and HMMER scanning [28]. The RAPSearch2 database was created from the protein sequences listed at the CAZy website [18, 47] (access date: 2014.09.18) while the profile HMMs were downloaded from dbCAN [76] (version: dbCAN-fam-HMMs.txt.v3). The outputs of these two program runs were compared and only their intersections were kept (i.e., loci confirmed by both methods). In case of conflicting family assignments, the RAPSearch2 results were preferred.

Overall, in its genome *F. rivuli* DSM 21788^T^ possess a variety of carbohydrate active enzymes including 94 glycoside hydrolases (GH) belonging to 31 families, 11 carbohydrate binding modules (CBM) belonging to 7 families, 13 carbohydrate esterases (CE) belonging to 8 families, one polysaccharide lyase of family 11 (PL11) and 37 glycosyl transferases belonging to 11 families (Table 8 and Additional file 1: Table S4). The carbohydrate esterases CE2, CE6, CE7, CE12 might act as carboxylic-ester hydrolases (EC 3.1.1.-) and the carbohydrate esterases CE11, CE14 as linear amides (EC 3.5.1.-). The genome of strain DSM 21788^T^ comprised a set of four GH5 and three GH51, for the potential hydrolysis of various cellulose or xylan polysaccharides. The absence of GH50, GH86 (agarose hydrolysis), GH18, GH19, GH20 (chitin hydrolysis) and a gene for alginate lyase (EC 4.2.2.3) corroborate the results of Ali et al. [1] that *F. rivuli* can not hydrolyze agarose, chitin and alginate, respectively. *F. rivuli* is equipped with one GH1, five GH5 and three GH30 as potential *β*-glucosidases and was shown to utilize cellobiose (d-Glc-*β*(1 → 4)-d-Glc) but not cellulose [1]. Gentobiose (d-Glc-*β*(1 → 6)-d-Glc) utilization and *β*-galactosidase activity was shown for *F. rivuli* [1] which has one GH1, fifteen GH2, eleven GH3 and one GH42 encoded in its genome. Starch was hydrolyzed by *F. rivuli* [1] presumably by enzyme activity of the four GH13 (*α*-amylase) and trehalose [1] by four GH13, one GH37, one GH65 (trehalase). The products of starch hydrolysis, maltose and d-glucose, can be utilized by *F. rivuli* [1]. Melibiose (d-Gal-*α*(1 → 6)-d-Glc) was metabolized by *F. rivuli* and *α*-galactosidase activity was confirmed [1], which might be mediated by the two GH27, two GH36 and five GH97.

**Table 8:** Carbohydrate active enzymes (CAZy) in the genome of strain DSM 21788^T^

CAZy family	GH1	GH2	GH3	GH5	GH13	GH16	GH23
Counts	1	15	11	4	4	2	2
CAZy family	GH25	GH27	GH28	GH29	GH30	GH31	GH36
Counts	1	2	5	2	3	5	2
CAZy family	GH37	GH39	GH42	GH43	GH51	GH65	GH73
Counts	1	3	1	11	3	1	1
CAZy family	GH78	GH88	GH92	GH95	GH97	GH105	GH106
Counts	1	1	2	3	5	4	2
CAZy family	GH127	GH130	GH^a^				
Counts	1	2	3				
CAZy family	GT2	GT4	GT5	GT9	GT19	GT20	GT28
Counts	13	10	1	3	1	1	1
CAZy family	GT30	GT41	GT51	GT^a^			
Counts	1	1	4	1			
CAZy family	CBM2	CBM10	CBM13	CBM32	CBM35	CBM50	CBM57
Counts	1	1	1	1	2	4	1
CAZy family	CE2	CE4	CE6	CE7	CE11	CE12	CE14
Counts	1	1	1	1	1	3	2
CAZy family	CE^a^		PL11				
Counts	3		1				

^a^genes attributed to an enzyme class, but not to a family

### Polysaccharide utilization loci

Members of flavobacteria were frequently found in aquatic habitats and play a pivotal role in the remineralization of complex organic matter [24, 39]. The coincidence of (i) a preference for polymeric substrates [39], (ii) the occurrence during algal blooms [62, 70] and (iii) the organization of genes involved in polysaccharide decomposition in polysaccharide utilization loci (PUL) [16, 67], suggests a specialization of *Flavobacteriia* members towards the utilization of complex organic matter.

**Fig. 4:**
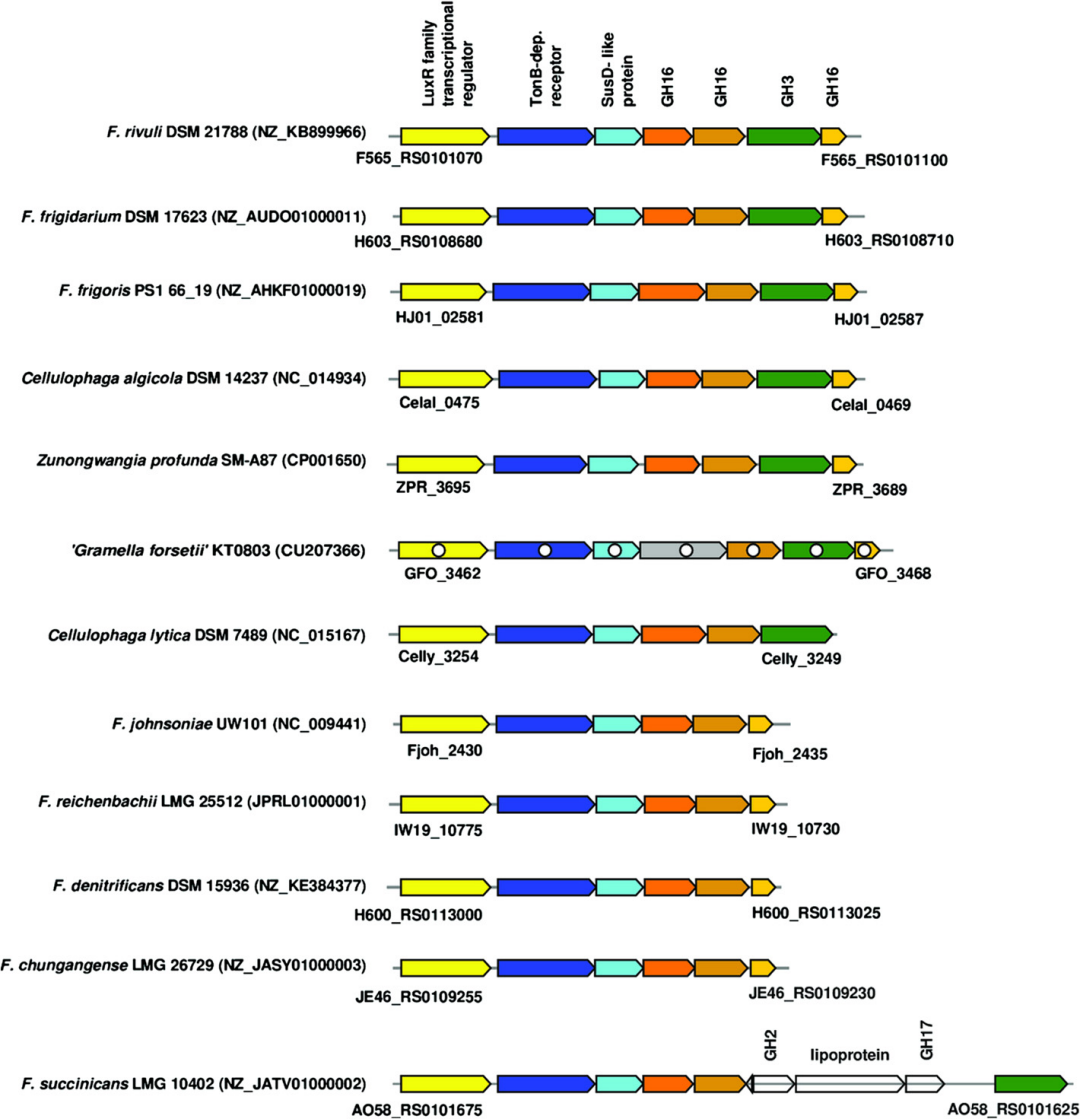
Synteny between a potentially laminarin-specific PUL of *F. rivuli* DSM 21788^T^ and other *Flavobacteriaceae*. Open circles indicate genes which were specifically expressed by ‘*Gramella forsetii*’ KT0803 when grown on laminarin, as shown by Kabisch et al. [38]. Locus tags are given below both the first and last gene of the loci. Accession numbers in brackets are GenBank accession numbers of the corresponding contig. Investigation of syntenic loci was done using MultiGeneBlast [52]. A description of glycoside hydrolase families (GH) can be seen at the CAZy homepage [18, 47]

**Fig. 5:**
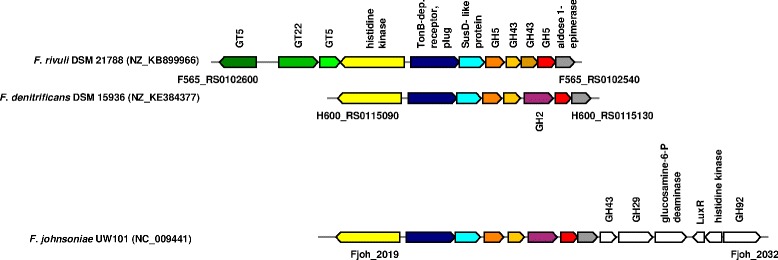
Synteny between a PUL of *F. rivuli* DSM 21788^T^, *F. denitrificans* DSM 15936^T^ and *F. johnsoniae* UW101^T^. Locus tags are given below both the first and last gene of the loci. Accession numbers in brackets are GenBank accession numbers of the corresponding contig. Investigation of syntenic loci was done using MultiGeneBlast [52]. A description of glycoside hydrolase families (GH) and glycoside transferase families (GT) can be seen at the CAZy homepage [18, 47]

**Fig. 6:**
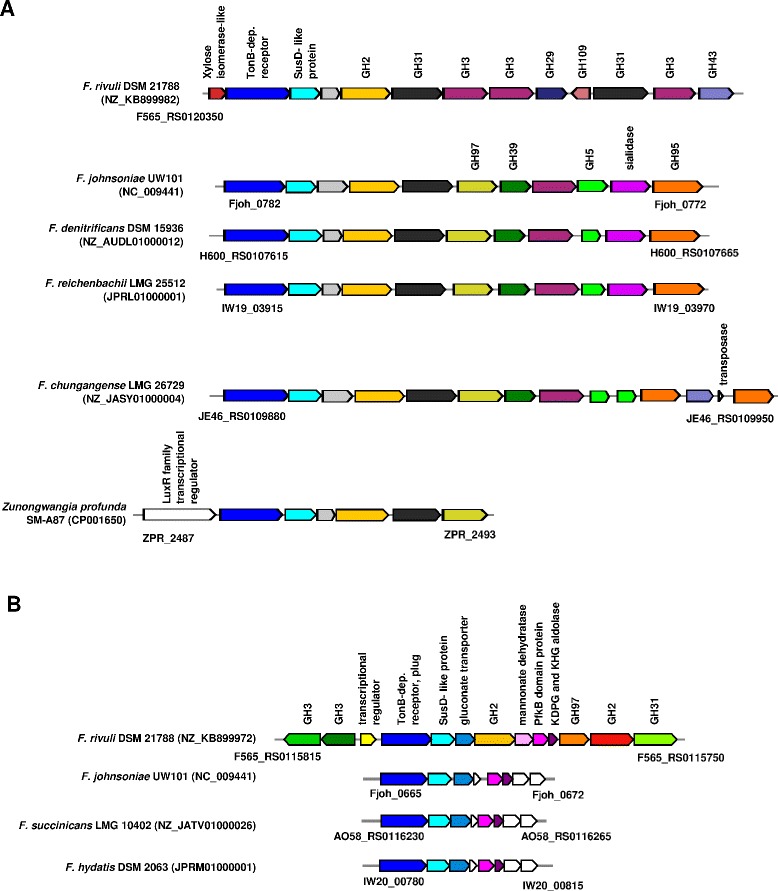
Two PUL of *F. rivuli* DSM 21788^T^ with low synteny (**a**, **b**) to PUL of other *Flavobacterium* members, potentially mediating the decomposition of hemicellulose or xylose. Locus tags are given below both the first and last gene of the loci. Accession numbers in brackets are GenBank accession numbers of the corresponding contig. Investigation of syntenic loci was done using MultiGeneBlast [52]. A description of glycoside hydrolase families (GH) can be seen at the CAZy homepage [18, 47]

**Fig. 7:**
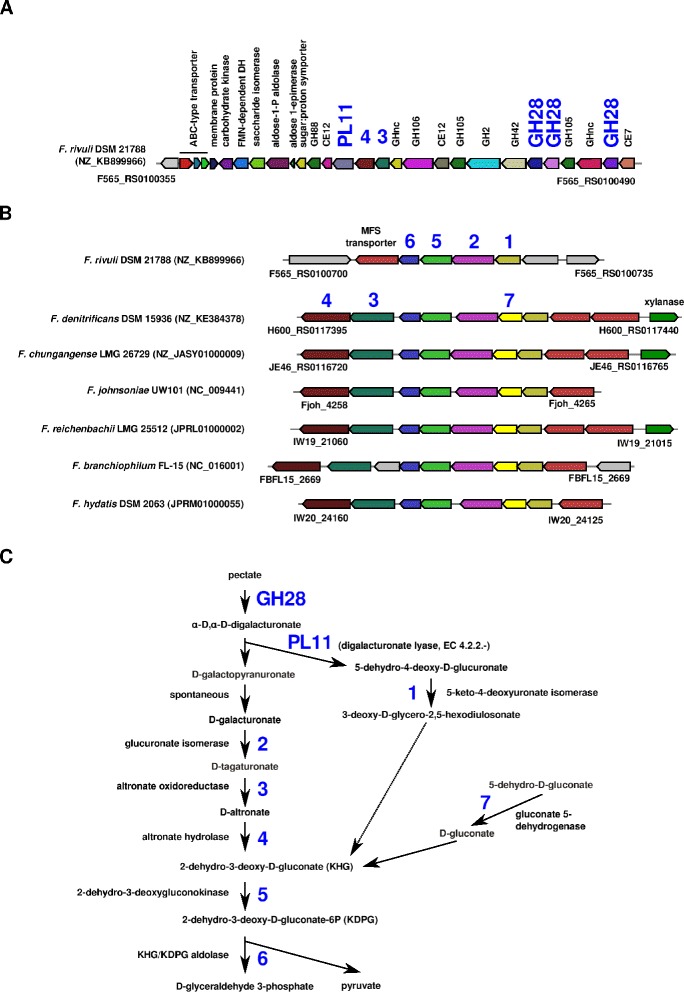
Polygalacturonate decomposition potential in F. rivuli DSM 21788T. **a** The potentially polygalacturonate specific PUL was found exclusively in F. rivuli DSM 21788T. **b** Genes for the catabolism of d-galactopyranuronate are colocalized in a gene cluster syntenic between Flavobacterium members. **c** Enzymes of the pectate decomposition and catabolism pathway. Bold blue numbers indicate the position of enzymes in the pectate catabolism pathway **c** and their corresponding genes in the gene clusters **a**, **b**. Genes in gray encode for hypothetical proteins. Locus tags are given below both the first and last gene of the loci. Accession numbers in brackets are GenBank accession numbers of the corresponding contig. Investigation of syntenic loci was done using MultiGeneBlast [52]. Investigation of pectin degradation pathway was done using the MetaCyc homepage [19]. A description of glycoside hydrolase families (GH) can be seen at the CAZy homepage [18, 47]

In *F. rivuli* DSM 21788^T^ four PULs were identified consisting of a TonB-dependent receptor, a SusD-like protein and a series of carbohydrate active enzymes (Figs. 4, 5, 6 and 7). The synteny between the identified PULs and 40 currently available *Flavobacteriaceae* genomes were investigated using MultiGeneBlast [52]. Figure 4 shows one of the PULs being conserved between some strains from the genera *Flavobacterium*, *Cellulophaga*, *Gramella* and *Zunongwangia*. Kabisch et al. [38] showed that proteins of the same PUL in ‘*Gramella forsetii*’ KT0803 were specifically expressed when grown on laminarin. The second PUL comprised of three glycosyl transferases, two GH5 and GH43 was found also in *F. denitrificans* DSM 15936^T^ and *F. johnsoniae* UW101 [50], but with an additional GH2 (Fig. 5). Two further PULs comprised combinations of GH2, GH3, GH31, GH97 and other glycoside hydrolases and were only partially identical with PULs of other *Flavobacterium* members (Fig. 6a and b). These PULs potentially enable *F. rivuli* to decompose hemicellulose or xylose.

In addition to the PULs, *F. rivuli* DSM 21788^T^ had one large operon-like structure comprising a set of 11 glycoside hydrolases, 3 carbohydrate esterases, one polysaccharide lyase (Fig. 7a), notably three GH28s (exo-poly-*α*-d-galacturonosidase) and a PL11 (digalacturonate lyase) for the decomposition of a pectate-like polysaccharide (polygalacturonate). Acetyl groups may be split of by CE7 (acetyl xylan esterase) and CE12 (rhamnogalacturonan acetylesterase). Interestingly, this operon additionally includes an altronate hydrolase and an oxidoreductase, which are part of the d-galactopyranuronate catabolic pathway (Fig. 7c), as well as two transporters, an aldose epimerase, a dehydrogenase and a kinase, which may mediate the catabolism of side-chain saccharides such as d-xylose, d-mannose and d-arabinose. In other *Flavobacterium* species, genes of the d-galactopyranuronate catabolic pathway are all co-located in loci which are syntenic with a gene cluster in *F. rivuli* (Fig. 7b). However, the gene cluster in *F. rivuli* did not contain the altronate hydrolase and oxidoreductase. Conclusively, the absence of the two genes of the d-galactopyranuronate catabolic pathway, and thus the ability to utilize polygalacturonate, was possibly compensated by the large CAZy-rich gene cluster.

## Conclusion

The high-quality draft genome sequence of the Gram-negative, non-motile *F. rivuli* WB 3.3-2^T^ (=DSM 21788^T^) isolated from a spring of a hard water rivulet provided new insights into the polysaccharide-decomposition potential of freshwater *Flavobacteriaceae*. *F. rivuli* belongs to a group of deep-branching species within the genus *Flavobacterium* that might be more closely related to the genus *Myroides* than to the type species of *Flavobacterium**,**F. aquatile*. The present data points towards an unsatisfactory taxonomy irrespective of which interpretation one follows and is largely a result of publishing new species in the genus *Flavobacterium* without taking into consideration a wider range of species in that genus or including members of the genus *Myroides* as well as publishing new species within the genus *Myroides* without taking a larger number of species from the genus *Flavobacterium* into consideration (including the type species). At the same time all evaluations are primarily based on “phylogenetic data” (i.e., gene sequence data) and genera are often poorly delineated. At first glance it does not appear that this approach will resolve this issue. Bernardet et al. [12] mentioned the clustering of *F. rivuli* among other *Flavobacterium* species in groups or possible new genera which have 16S rRNA gene sequence identities below 93 % with the type species *F. aquatile* of the genus. However, the potentially new genera could not be delineated because different procedures or culture conditions were used to describe common features [12].

The problem of an essentially unresolved backbone in the 16S rRNA gene sequence phylogeny of the *Flavobacteriaceae* (see above) will most probably be overcome in the near future with the foreseeable increase of publicly available draft genome sequences from large scale projects such as GEBA, which will enable us to infer whole genome sequence based phylogenies with a significantly higher statistical support for the branching topology using genome-based inference methods [54].

The genome of strain *F. rivuli* WB 3.3-2^T^ (DSM 21788^T^) comprised 4.48 Mbp on 23 scaffolds and was sequenced as part of the ***G****enomic****E****ncyclopedia of****B****acteria and****A****rchaea* project. The genome encoded for a great variety of 151 carbohydrate active enzymes and 177 peptidases. The four identified polysaccharide-utilization loci may enable strain WB 3.3-2^T^ to decompose laminarin, hemicellulose and xylose. One gene cluster was identified that may enable strain WB 3.3-2^T^ to decompose pectate-like polysaccharides. This genome in combination with other genomes of the *Flavobacteriaceae* will give further insights into the evolution and genetic potential of bacteria succeeding in substrate-related niches during polysaccharide decomposition in marine and freshwater habitats.

## Additional file

Additional file 1: Table S1. Accession numbers of 16S rRNA gene sequences of *Flavobacterium*, *Myroides*, *Capnocytophaga* and *Coenonia* type strains used for generating the phylogenetic tree (Fig. 1) and the histogram (Fig. 2). **Table S2.** Peptidases or homologues in the genome of *F. rivuli* DSM 21788^T^. **Table S3.** Simple peptidases inhibitors in the genome of *F. rivuli* DSM 21788^T^. **Table S4.** Carbohydrate active enzymes (CAZymes) in the genome of *F. rivuli* DSM 21788^T^. **Table S5.** Sulfatases in the genome of *F. rivuli* DSM 21788^T^.
