# Supplementary material for: High quality draft genome sequence of Flavobacterium rivuli type strain WB 3.3-2T (DSM 21788T), a valuable source of polysaccharide decomposing enzymes
Source: Stand Genomic Sci. 2015 Jul 30;10:46. doi: 10.1186/s40793-015-0032-y (PMC4572689; doi:10.1186/s40793-015-0032-y)
Supplement: Additional file 1: Table S1. — Accession numbers of 16S rRNA gene sequences of Flavobacterium, Myroides, Capnocytophaga and Coenonia type strains used for generating the phylogenetic tree (Fig. 1) and the histogram (Fig. 2). Table S2. Peptidases or homologues in the genome of F. rivuli DSM 21788T. Table S3. Simple peptidases inhibitors in the genome of F. rivuli DSM 21788T. Table S4. Carbohydrate active enzymes (CAZymes) in the genome of F. rivuli DSM 21788T. Table S5. Sulfatases in the genome of F. rivuli DSM 21788T. [file 40793_2015_32_MOESM1_ESM.pdf]

High quality draft genome sequence of *Flavobacterium rivuli* type strain WB 3.3-2^T (DSM 21788^T), a valuable source of polysaccharide decomposing enzymes

Richard L. Hahnke[1,*], Erko Stackebrandt[1], Jan P. Meier-Kolthoff[1], Brian J. Tindall[1], Sixing Huang[1], Manfred Rohde[2], Alla Lapidus[3,4], James Han[5], Stephan Trong[5] ,Matthew Haynes[5], T.B.K. Reddy[5], Marcel Huntemann[5], Amrita Pati[5], Natalia N. Ivanova[5], Konstantinos Mavromatis[5], Victor Markowitz[6], Tanja Woyke[5], Markus Göker[1], Nikos C. Kyrpides[5,7] and Hans-Peter Klenk[8]

[1] Leibniz Institute DSMZ – German Collection of Microorganisms and Cell Cultures, Inhoffenstraße 7B, Braunschweig, Germany
[2] Helmholtz Centre for Infection Research, Inhoffenstraße 7, Braunschweig, Germany
[3] St. Petersburg State University, St. Petersburg, Russia
[4] Algorithmic Biology Lab, St. Petersburg Academic University, St.Petersburg, Russia
[5] DOE Joint Genome Institute, Walnut Creek, California, USA
[6] Biological Data Management and Technology Center, Lawrence Berkeley National Laboratory, Berkeley, California, USA
[7] School of Biology, King Abdulaziz University, Jeddah, Saudi Arabia
[8] School of Biology, Newcastle University, Newcastle upon Tyne, UK

*Corresponding author: Richard L. Hahnke

Supporting Information Text

Table S1. Accession numbers of 16S rRNA gene sequences of Flavobacterium, Myroides, Capnocytophaga and Coenonia type strains used for generating the phylogenetic tree (Figure 1) and the histogram (Figure 2).

Name	Acc. number
Flavobacterium aciduliphilum JJ013 ^{T}	JN712178
Flavobacterium algicola TC2 ^{T}	AB455265
Flavobacterium anatoliense MK3 ^{T}	JF825522
Flavobacterium anhuiense D3 ^{T}	EU046269
Flavobacterium antarcticum AT1026^T	AY581113
Flavobacterium aquatile DSM1132 ^{T}	AM230485
Flavobacterium aquidurense WB1.1-56 ^{T}	AM177392
Flavobacterium araucananum LM-19-Fp ^{T}	FR774916
Flavobacterium banpakuense 15F3 ^{T}	GQ281770
Flavobacterium beibuense F44-8 ^{T}	GQ245972
Flavobacterium caeni LM5 ^{T}	EU313814
Flavobacterium cauense R2A-7 ^{T}	EU521691
Flavobacterium ceti 454-2 ^{T}	AM292800
Flavobacterium cheniae NJ-26 ^{T}	EF407880
Flavobacterium cheonanense ARSA-108 ^{T}	GU295968
Flavobacterium cheonhonense ARSA-15 ^{T}	GU295972
Flavobacterium chilense LM-09-Fp ^{T}	FR774915
Flavobacterium chungangense CJ7 ^{T}	EU924275
Flavobacterium chungbukense CS100 ^{T}	HM627539
Flavobacterium chungnamense ARSA-103 ^{T}	GU295971
Flavobacterium columnare IFO15943 ^{T}	AB078047
Flavobacterium compostarboris 15C3 ^{T}	GQ281769
Flavobacterium croceum EMB47 ^{T}	DQ372982
Flavobacterium cucumis R2A45-3 ^{T}	EF126993
Flavobacterium cutihirudinis E89 ^{T}	JX966231
Flavobacterium daejeonense GH1-10 ^{T}	DQ222427
Flavobacterium dankookense ARSA-19 ^{T}	GU295970
Flavobacterium defluvii EMB117 ^{T}	DQ372986
Flavobacterium degerlachei LMG21915 ^{T}	AJ557886
Flavobacterium denitrificans ED5 ^{T}	AJ318907
Flavobacterium dongtanense LW30 ^{T}	GU073293
Flavobacterium enshiense DK69 ^{T}	JN790956
Flavobacterium filum EMB34 ^{T}	DQ372981
Flavobacterium flevense DSM1076 ^{T}	AM230486
Flavobacterium fluvii H7 ^{T}	EU109724
Flavobacterium fontis MIC3010 ^{T}	JN873147
Flavobacterium frigidarium A2i ^{T}	AF162266
Flavobacterium frigidimaris KUC-1 ^{T}	AB183888
Flavobacterium frigoris LMG21922 ^{T}	AJ557887
Flavobacterium fryxellicola LMG22022 ^{T}	AJ811961

Continued on next page.

Table S1. (continued)

Name	Acc. number
Flavobacterium gelidilacus R-8899 T	AJ440996
Flavobacterium gillisiae IC001 T	U85889
Flavobacterium ginsengisoli DCY54 T	HM776705
Flavobacterium ginsenosidimutans THG1 T	GU138377
Flavobacterium glaciei 0499 T	DQ515962
Flavobacterium glycines Gm-149 T	EU672803
Flavobacterium granuli Kw05 T	AB180738
Flavobacterium haoranii LQY-7 T	GQ988780
Flavobacterium hercynium WB4.2-33 T	AM265623
Flavobacterium hibernum ATCC51468 T	L39067
Flavobacterium hydatis M58764 T	CYTRR16SA
Flavobacterium indicum GPTSA100-9 T	AY904351
Flavobacterium johnsoniae DSM2064 T	AM230489
Flavobacterium jumunjinense HME7102 T	JF317279
Flavobacterium koreense ARSA-42 T	GU295967
Flavobacterium kyungheense THG-107 T	JN196130
Flavobacterium lacus NP180 T	KC969641
Flavobacterium limicola ST-82 T	AB075230
Flavobacterium limnosediminis JC2902 T	JQ928688
Flavobacterium lindanitolerans IP10 T	EF424395
Flavobacterium longum YIT 12745 T	AB795014
Flavobacterium macrobrachii an-8 T	FJ593904
Flavobacterium marinum SW105 T	JN867054
Flavobacterium micromati LMG21919 T	AJ557888
Flavobacterium myungsuense HMD1033 T	GQ148878
Flavobacterium noncentrifugens R-HLS-17 T	JQ002654
Flavobacterium omnivorum AS1.2747 T	AF433174
Flavobacterium oncorhynchi 631-08 T	FN669776
Flavobacterium pectinovorum DSM6368 T	AM230490
Flavobacterium phragmitis BLN_2 T	GU564236
Flavobacterium piscis 412R-09 T	HE612101
Flavobacterium plurextorum 1126-1H-08 T	HE612094
Flavobacterium ponti GSW-R14 T	GQ370387
Flavobacterium psychrolimnae LMG22018 T	AJ585428
Flavobacterium psychrophilum IFO15942 T	AB078060
Flavobacterium rakeshii FCS-5 T	JF830803
Flavobacterium reichenbachii WB3.2-61 T	AM177616
Flavobacterium resistens BD-b365 T	EF575563
Flavobacterium rivuli WB 3.3-2 T	AM934661
Flavobacterium saccharophilum DSM1811 T	AM230491
Flavobacterium saliperosum S13 T	DQ021903
Flavobacterium sasangense YC6274 T	EU423319

Continued on next page.

Table S1. (continued)

Name	Acc. number
Flavobacterium segetis AT1048 T	AY581115
Flavobacterium sinopsychrotolerans 0533 T	FJ654474
Flavobacterium soli DS-6 T	DQ178976
Flavobacterium squillarum CMJ-5 T	JQ029111
Flavobacterium subsaxonicum WB 4.1-42 T	AM934666
Flavobacterium succinicans DSM4002 T	AM230492
Flavobacterium suncheonense GH29-5 T	DQ222428
Flavobacterium swingsii WB2.3-68 T	AM934651
Flavobacterium tegetincola ACAM 602 T	U85887
Flavobacterium terrae R2A1-13 T	EF117329
Flavobacterium terrigena DS-20 T	DQ889724
Flavobacterium tiangeerense 0563 T	EU036219
Flavobacterium tilapiae Ruye-71 T	HQ111525
Flavobacterium ummariense DS-12 T	HQ329187
Flavobacterium urocaniciphilum YIT 12746 T	AB795015
Flavobacterium urumqiense Sr25 T	HQ436467
Flavobacterium weaverense AT1042 T	AY581114
Flavobacterium xanthum ACAM81 T	AF030380
Flavobacterium xinjiangense AS1.2749 T	AF433173
Flavobacterium xueshanense Sr22 T	HQ436466
Flavobacterium yanchengense hg T	JX548325
Flavobacterium yonginense HMD1001 T	GQ144413
Myroides marinus JS-08 T	GQ857652
Myroides odoratimimus CCUG39352 T	AJ854059
Myroides odoratus M58777 T	FVBRR16SG
Myroides pelagicus SM1 T	AB176662
Myroides phaeus MY15 T	GU253339
Myroides profundi D25 T	EU204978
Capnocytophaga canimorsus CIP103936 T	AY643075
Capnocytophaga cynodegmi CIP103937 T	AY643076
Capnocytophaga gingivalis ATCC33624 T	X67608
Capnocytophaga granulosa LMG16022T T	U41347
Capnocytophaga haemolytica ATCC51501 T	X97247
Capnocytophaga leadbetteri AHN8855 T	DQ009623
Capnocytophaga ochracea ATCC27872 T	U41350
Capnocytophaga sputigena ATCC33612 T	X67609
Coenonia anatina LMG14382 T	Y17612

Table S2. Peptidases or homologues in the genome of *F. rivuli* DSM 21788T.

Locus tag (G498_)	MEROPS family	Peptidase or homologue	Accession No.
RS0114125	A08	signal peptidase II (Flavobacterium psychrophilum)	WP_020213887.1
RS0110145	A28	family A28 unassigned peptidases (Joostella marina)	WP_020213113.1
RS0120275	C01B	subfamily C1B unassigned peptidases (Flavobacterium frigoris)	WP_020215083.1
RS0111850	C25	family C25 unassigned peptidases	WP_020213447.1
RS0103125	C26	imidazole glycerol phosphate synthase subunit HisH	WP_020211739.1
RS0103135	C26	imidazole glycerol phosphate synthase subunit HisH (Prochlorococcus marinus)	WP_020211741.1
RS0107820	C26	dihydro-orotase (N-terminal unit) (Ailuropoda melanoleuca)	WP_020212657.1
RS0107990	C26	CTP synthetase	WP_020212691.1
RS0108195	C26	GMP synthase	WP_020212732.1
RS0108340	C26	trp1 (Bacteroides thetaiotaomicron)	WP_020212761.1
RS0112920	C26	family C26 non-peptidase homologues (Flavobacterium psychrophilum)	WP_020213659.1
RS0115150	C26	family C26 unassigned peptidases (Leadbetterella byssophila)	WP_020214086.1
RS0100295	C40	family C40 unassigned peptidases (Flavobacterium johnsoniae)	WP_020211180.1
RS0107955	C40	spr peptidase (Thermodesulfobacterium sp. OPB45)	WP_020212684.1
RS0108135	C40	PgpA peptidase (Flavobacterium columnare)	WP_020212720.1
RS0107710	C44	glutamate synthase (alpha chain) precursor (Niastella koreensis)	WP_020212635.1
RS0108280	C44	AsnB protein (Flavobacterium frigoris)	WP_020212749.1
RS0109720	C44	glucosamine-fructose-6-phosphate aminotransferase	WP_020213031.1
RS0113855	C44	family C44 unassigned peptidases (Flavobacterium psychrophilum)	WP_020213837.1
RS0100655	C56	PfpI peptidase (Flavobacterium johnsoniae)	WP_020211253.1
RS0104520	C56	family C56 non-peptidase homologues (Methanoculleus marisnigri)	WP_020212009.1
RS0106930	C56	KIAA0361 protein ({Homo sapiens}-type) (Dyadobacter fermentans)	WP_020212480.1
RS0110340	C56	family C56 non-peptidase homologues (Pedobacter heparinus)	WP_026299990.1
RS0112675	C82	YafK protein	WP_020213612.1
RS0100515	M01	family M1 unassigned peptidases (Leeuwenhoekiella blandensis)	WP_020211225.1
RS0100940	M01	family M1 unassigned peptidases (Flavobacterium indicum)	WP_020211309.1

Continued on next page.

Table S2. (continued)

Locus tag (G498_)	MEROPS family	Peptidase or homologue	Accession No.
RS0102155	M01	family M1 unassigned peptidases (Flavobacterium indicum)	WP_020211544.1
RS0113460	M01	family M1 unassigned peptidases (Flavobacterium branchiophilum)	WP_020213764.1
RS0113590	M01	family M1 unassigned peptidases (Flavobacterium johnsoniae)	WP_020213790.1
RS0114215	M01	family M1 unassigned peptidases (Flavobacterium indicum)	WP_020213905.1
RS0110995	M03A	subfamily M3A unassigned peptidases (Flavobacterium branchiophilum)	WP_020213278.1
RS0112035	M03A	subfamily M3A unassigned peptidases (Flavobacterium johnsoniae)	WP_020213484.1
RS0119840	M12B	subfamily M12B unassigned peptidases (Weeksella virosa)	WP_020214999.1
RS0120655	M12B	subfamily M12B unassigned peptidases (Endoriftia persephone)	WP_026300221.1
RS0110570	M13	Zmp1 peptidase (Flavobacterium frigoris)	WP_020213197.1
RS0118665	M13	Zmp1 peptidase (Flavobacterium sp. CF136)	WP_020214766.1
RS0108800	M14B	subfamily M14B non-peptidase homologues (Dyadobacter fermentans)	WP_020212853.1
RS0111570	M14B	subfamily M14B non-peptidase homologues	WP_020213393.1
RS0115020	M14B	subfamily M14B non-peptidase homologues (Dokdonia donghaensis)	WP_020214060.1
RS0108840	M14B	subfamily M14B non-peptidase homologues (Dyadobacter fermentans)	WP_026299969.1
RS0114895	M14X	family M14 non-peptidase homologues (Flavobacterium columnare)	WP_020214037.1
RS0117400	M14X	family M14 non-peptidase homologues (Niastella koreensis)	WP_020214526.1
RS0102145	M14X	family M14 non-peptidase homologues (Polaribacter sp. MED152)	WP_026299831.1
RS0114315	M14X	family M14 non-peptidase homologues (Flavobacterium psychrophilum)	WP_026300066.1
RS0106290	M16B	At5g56730 peptidase (Kordia algicida)	WP_020212355.1
RS0106200	M16B	subfamily M16B non-peptidase homologues (Flavobacterium indicum)	WP_026299914.1
RS0106205	M16B	subfamily M16B non-peptidase homologues (Flavobacterium indicum)	WP_026299915.1
RS0117010	M19	family M19 non-peptidase homologues (Pedobacter agri)	WP_020214448.1
RS0100260	M20A	BT3549 putative peptidase (Flavobacterium johnsoniae)	WP_020211173.1
RS0115135	M20B	peptidase T (Myroides injenensis)	WP_020214083.1
RS0104820	M20C	Pep581 peptidase (Flavobacterium sp. CF136)	WP_020212069.1
RS0110840	M20F	subfamily M20F unassigned peptidases (Flavobacterium columnare)	WP_020213249.1
RS0100125	M20X	family M20 unassigned peptidases (Campylobacter jejuni)	WP_020211146.1

Continued on next page.

Table S2. (continued)

Locus tag (G498_)	MEROPS family	Peptidase or homologue	Accession No.
RS0107610	M23B	subfamily M23B unassigned peptidases (Polaribacter sp. MED152)	WP_020212614.1
RS0107625	M23B	subfamily M23B unassigned peptidases (Bizionia argentinensis)	WP_020212617.1
RS0109990	M23B	Mername-AA292 peptidase (Flavobacterium indicum)	WP_020213084.1
RS0114095	M23B	Mername-AA292 peptidase	WP_020213881.1
RS0119235	M23B	subfamily M23B non-peptidase homologues (Flavobacterium johnsoniae)	WP_020214879.1
RS0119530	M23B	Mername-AA292 peptidase (Lacinutrix sp. 5H-3-7-4)	WP_020214935.1
RS0119115	M23B	subfamily M23B non-peptidase homologues (Fibrobacter succinogenes)	WP_026300182.1
RS0119385	M23B	Mername-AA292 peptidase (Leeuwenhoekiella blandensis)	WP_026300189.1
RS0106270	M24A	methionyl aminopeptidase 1 (Flavobacterium psychrophilum)	WP_020212352.1
RS0114470	M24B	subfamily M24B unassigned peptidases (Flavobacterium johnsoniae)	WP_020213956.1
RS0100665	M28A	subfamily M28A unassigned peptidases (Psychroflexus torquis)	WP_026299799.1
RS0111885	M28E	subfamily M28E unassigned peptidases (Flavobacterium johnsoniae)	WP_020213454.1
RS0101805	M38	family M38 non-peptidase homologues (Agrobacterium tumefaciens)	WP_020211476.1
RS0102650	M38	family M38 non-peptidase homologues (Flavobacterium columnare)	WP_020211643.1
RS0102815	M38	family M38 non-peptidase homologues (Methylobacterium nodulans)	WP_020211677.1
RS0108075	M38	family M38 non-peptidase homologues (Flavobacterium johnsoniae)	WP_020212708.1
RS0111505	M38	family M38 non-peptidase homologues (Flavobacterium indicum)	WP_020213380.1
RS0113615	M38	family M38 non-peptidase homologues (Kangiella koreensis)	WP_020213795.1
RS0115650	M41	Afg3-like protein 2 (Flavobacterium psychrophilum)	WP_020214185.1
RS0103580	M42	family M42 unassigned peptidases (Flavobacterium psychrophilum)	WP_020211829.1
RS0118735	M43B	subfamily M43B unassigned peptidases (Dyadobacter fermentans)	WP_020214780.1
RS0106825	M48C	subfamily M48C unassigned peptidases (Flavobacterium johnsoniae)	WP_020212459.1
RS0108970	M50B	subfamily M50B unassigned peptidases (Flavobacterium psychrophilum)	WP_020212884.1
RS0112555	M61	family M61 unassigned peptidases (Flavobacterium johnsoniae)	WP_026300036.1
RS0110705	M75	imelysin (Flavobacterium psychrophilum)	WP_020213224.1
RS0110695	M75	family M75 unassigned peptidases (Spirosoma linguale)	WP_026299998.1

Continued on next page.

Table S2. (continued)

Locus tag (G498_)	MEROPS family	Peptidase or homologue	Accession No.
RS0102165	M79	family M79 unassigned peptidases (Cellulophaga algicola)	WP_020211546.1
RS0109730	M90	family M90 unassigned peptidases (Leeuwenhoekiella blandensis)	WP_020213033.1
RS0104855	M96	family M96 non-peptidase homologues (Flavobacterium rivuli)	WP_020212076.1
RS0118690	M96	family M96 non-peptidase homologues (Flavobacterium rivuli)	WP_020214771.1
RS0104305	N11	family N11 unassigned peptide lyases	WP_020211973.1
RS0103855	S01C	DegQ peptidase (Flavobacterium indicum)	WP_020211883.1
RS0113555	S01D	subfamily S1D unassigned peptidases (Psychroflexus torquis)	WP_020213783.1
RS0104100	S08A	subfamily S8A unassigned peptidases	WP_020211932.1
RS0109335	S08A	subfamily S8A non-peptidase homologues (Fluviicola taffensis)	WP_020212958.1
RS0109365	S08A	subfamily S8A non-peptidase homologues (Fluviicola taffensis)	WP_020212964.1
RS0109645	S08A	subfamily S8A unassigned peptidases (Flavobacterium psychrophilum)	WP_020213016.1
RS0119660	S08A	subfamily S8A non-peptidase homologues (Flavobacterium columnare)	WP_020214961.1
RS0102665	S09A	oligopeptidase B (Flavobacterium indicum)	WP_020211646.1
RS0119200	S09A	prolyl oligopeptidase (Ignavibacterium album)	WP_020214872.1
RS0120235	S09A	prolyl oligopeptidase (Flavobacterium johnsoniae)	WP_020215075.1
RS0105615	S09B	dipeptidyl-peptidase 4 (bacteria-type 2) (Flavobacterium branchiophilum)	WP_020212225.1
RS0115940	S09B	prolyl tripeptidyl peptidase (Flavobacterium columnare)	WP_020214242.1
RS0103240	S09C	subfamily S9C unassigned peptidases (Dyadobacter fermentans)	WP_020211762.1
RS0107835	S09C	dpf-6 g.p. (Porphyromonas gingivalis)	WP_020212660.1
RS0120270	S09C	dipeptidyl-peptidase 5 ({Porphyromonas gingivalis}) (Flavobacterium columnare)	WP_020215082.1
RS0100490	S09C	subfamily S9C unassigned peptidases (Bacteroides vulgatus)	WP_026299795.1
RS0100660	S09X	family S9 non-peptidase homologues (Bdellovibrio bacteriovorus)	WP_020211254.1
RS0101725	S09X	family S9 non-peptidase homologues (Acinetobacter baumannii)	WP_020211461.1
RS0102705	S09X	acetylcholinesterase (Chitinophaga pinensis)	WP_020211655.1
RS0102850	S09X	family S9 unassigned peptidases (Chitinophaga pinensis)	WP_020211684.1
RS0103010	S09X	family S9 non-peptidase homologues	WP_020211716.1

Continued on next page.

Table S2. (continued)

Locus tag (G498_)	MEROPS family	Peptidase or homologue	Accession No.
RS0103155	S09X	family S9 unassigned peptidases (Acaryochloris marina)	WP_020211745.1
RS0103940	S09X	family S9 non-peptidase homologues (Zunongwangia profunda)	WP_020211900.1
RS0106820	S09X	family S9 non-peptidase homologues (Flavobacterium psychrophilum)	WP_020212458.1
RS0107325	S09X	family S9 unassigned peptidases (Haliscomenobacter hydrossis)	WP_020212558.1
RS0107405	S09X	family S9 unassigned peptidases (Chitinophaga pinensis)	WP_020212574.1
RS0108085	S09X	YpfH protein (Flavobacterium johnsoniae)	WP_020212710.1
RS0109945	S09X	family S9 non-peptidase homologues (Spirosoma linguale)	WP_020213075.1
RS0112540	S09X	family S9 non-peptidase homologues (Methanosarcina acetivorans)	WP_020213585.1
RS0115295	S09X	family S9 non-peptidase homologues (Methylobacterium chloromethanicum)	WP_020214114.1
RS0116685	S09X	family S9 unassigned peptidases (Flavobacterium johnsoniae)	WP_020214384.1
RS0119310	S09X	family S9 non-peptidase homologues (Flavobacterium johnsoniae)	WP_020214893.1
RS0119845	S09X	family S9 non-peptidase homologues (Capnocytophaga ochracea)	WP_020215000.1
RS0102915	S09X	family S9 non-peptidase homologues (Acidovorax avenae)	WP_026299846.1
RS0103150	S09X	family S9 non-peptidase homologues (Gramella forsetii)	WP_026299852.1
RS0104645	S09X	family S9 non-peptidase homologues (Flavobacterium johnsoniae)	WP_026299885.1
RS0105195	S09X	family S9 non-peptidase homologues (Acidovorax ebreus)	WP_026299894.1
RS0120220	S09X	family S9 non-peptidase homologues (Spirosoma linguale)	WP_026300204.1
RS0113550	S11	family S11 unassigned peptidases (Runella slithyformis)	WP_020213782.1
RS0100070	S12	family S12 unassigned peptidases (Flavobacterium sp. CF136)	WP_020211136.1
RS0101000	S12	family S12 unassigned peptidases	WP_020211321.1
RS0102030	S12	family S12 unassigned peptidases	WP_020211519.1
RS0110030	S12	family S12 unassigned peptidases (Flavobacterium rivuli)	WP_020213092.1
RS0113735	S12	family S12 non-peptidase homologues (Flavobacterium rivuli)	WP_020213817.1
RS0119650	S12	family S12 unassigned peptidases (Flavobacterium indicum)	WP_020214959.1
RS0105745	S14	family S14 non-peptidase homologues (Atopobium rimae)	WP_020212248.1
RS0111740	S14	peptidase Clp (type 1) (Flavobacterium johnsoniae)	WP_020213426.1

Continued on next page.

Table S2. (continued)

Locus tag (G498_)	MEROPS family	Peptidase or homologue	Accession No.
RS0114495	S14	family S14 non-peptidase homologues (Glaciecola sp. 4H-3-7+YE-5)	WP_020213961.1
RS0106845	S16	Lon peptidase (type 4) ()	WP_020212463.1
RS0109680	S16	family S16 non-peptidase homologues (Flavobacterium psychrophilum)	WP_020213023.1
RS0116330	S16	family S16 non-peptidase homologues (Flavobacterium branchiophilum)	WP_020214315.1
RS0114785	S24	family S24 unassigned peptidases (Bacteroides xylanisolvens)	WP_020214017.1
RS0116630	S24	family S24 non-peptidase homologues (Leadbetterella byssophila)	WP_020214373.1
RS0118890	S24	UmuD protein (Flavobacterium sp. CF136)	WP_020214811.1
RS0101510	S24	family S24 unassigned peptidases (Bacteroides xylanisolvens)	WP_026299820.1
RS0107260	S26A	subfamily S26A unassigned peptidases (Zobellia galactanivorans)	WP_020212547.1
RS0100775	S33	family S33 unassigned peptidases (Spirosoma linguale)	WP_020211276.1
RS0101765	S33	family S33 non-peptidase homologues (Acidobacterium capsulatum)	WP_020211468.1
RS0102875	S33	family S33 unassigned peptidases (Dyadobacter fermentans)	WP_020211689.1
RS0102905	S33	family S33 unassigned peptidases (Dyadobacter fermentans)	WP_020211695.1
RS0104255	S33	family S33 unassigned peptidases (Spirosoma linguale)	WP_020211963.1
RS0105350	S33	family S33 unassigned peptidases (Flavobacterium psychrophilum)	WP_020212173.1
RS0106470	S33	family S33 non-peptidase homologues (Flavobacterium johnsoniae)	WP_020212388.1
RS0107755	S33	family S33 unassigned peptidases (Flavobacterium johnsoniae)	WP_020212644.1
RS0109000	S33	family S33 non-peptidase homologues (Dyadobacter fermentans)	WP_020212890.1
RS0109495	S33	family S33 unassigned peptidases (Flavobacterium johnsoniae)	WP_020212988.1
RS0113280	S33	family S33 unassigned peptidases (Flavobacterium johnsoniae)	WP_020213730.1
RS0114480	S33	SCO7095-type peptidase (Flavobacterium psychrophilum)	WP_020213958.1
RS0115180	S33	family S33 non-peptidase homologues (Burkholderia phytofirmans)	WP_020214092.1
RS0115285	S33	family S33 non-peptidase homologues (Streptomyces clavuligerus)	WP_020214112.1
RS0116595	S33	family S33 unassigned peptidases (Cellulophaga algicola)	WP_020214366.1
RS0119935	S33	family S33 unassigned peptidases (Dyadobacter fermentans)	WP_020215017.1
RS0105570	S41A	C-terminal processing peptidase-1 (Saprospira grandis)	WP_020212216.1

Continued on next page.

Table S2. (continued)

Locus tag (G498_)	MEROPS family	Peptidase or homologue	Accession No.
RS0108695	S41A	subfamily S41A unassigned peptidases (Flavobacterium columnare)	WP_020212832.1
RS0109510	S41A	subfamily S41A unassigned peptidases (Zunongwangia profunda)	WP_020212991.1
RS0109915	S41A	C-terminal processing peptidase-3	WP_020213069.1
RS0115885	S41A	C-terminal processing peptidase-1 (Flavobacterium johnsoniae)	WP_026300102.1
RS0119140	S41A	CtpC peptidase (Flavobacterium columnare)	WP_026300183.1
RS0111705	S46	family S46 unassigned peptidases (Imtechella halotolerans)	WP_020213419.1
RS0105170	S46	family S46 unassigned peptidases (Flavobacterium johnsoniae)	WP_026299893.1
RS0111685	S49B	BSn5_05605 g.p. (Flavobacterium frigoris)	WP_020213415.1
RS0113660	S51	alpha-aspartyl dipeptidase (eukaryote) (Flavobacterium sp. CF136)	WP_020213803.1
RS0120830	S51	family S51 unassigned peptidases (Niastella koreensis)	WP_020215184.1
RS0105075	S54	family S54 unassigned peptidases (Flavobacterium johnsoniae)	WP_020212118.1
RS0107235	S54	RhoII peptidase ({Haloferax volcanii}) and similar (Flavobacterium columnare)	WP_020212542.1
RS0101020	S54	family S54 unassigned peptidases (Flavobacterium psychrophilum)	WP_026299806.1
RS0107240	S54	family S54 unassigned peptidases (Flavobacterium columnare)	WP_026299935.1
RS0102710	S66	murein tetrapeptidase LD-carboxypeptidase (Flavobacterium johnsoniae)	WP_020211656.1
RS0119825	S66	family S66 unassigned peptidases (Flavobacterium johnsoniae)	WP_026300197.1
RS0120835	T02	At3g16150 (Arthrospira platensis)	WP_020215185.1
RS0109095	U32	family U32 unassigned peptidases (Elizabethkingia anophelis)	WP_020212909.1
RS0113940	U32	family U32 unassigned peptidases (Chthonomonas calidirosea)	WP_020213852.1
RS0118110	U73	small protease (Pseudomonas aeruginosa)	WP_020214659.1

Table S3. Simple peptidases inhibitors in the genome of *F. rivuli* DSM 21788[T].

Locus tag ()	MEROPS family	Simple peptidase inhibitors	Accession No.
RS0101625	I39	family I39 unassigned peptidase inhibitor homologues (Pedobacter heparinus)	WP_020211442.1
RS0103070	I39	family I39 unassigned peptidase inhibitors (Chitinophaga pinensis)	WP_020211728.1
RS0103565	I39	family I39 unassigned peptidase inhibitors (Chitinophaga pinensis)	WP_020211826.1
RS0115795	I39	family I39 unassigned peptidase inhibitors (Spirosoma linguale)	WP_020214213.1
RS0116695	I39	family I39 unassigned peptidase inhibitor homologues (Pedobacter heparinus)	WP_020214385.1
RS0117265	I39	family I39 unassigned peptidase inhibitor homologues (Dyadobacter fermentans)	WP_020214499.1
RS0118045	I39	family I39 unassigned peptidase inhibitor homologues (Chitinophaga pinensis)	WP_020214646.1
RS0119700	I39	family I39 unassigned peptidase inhibitors (Pirellula staleyi)	WP_020214969.1
RS0120625	I39	family I39 unassigned peptidase inhibitors (Chitinophaga pinensis)	WP_020215148.1
RS0120785	I39	family I39 unassigned peptidase inhibitors (Spirosoma linguale)	WP_020215175.1
RS0101075	I39	family I39 unassigned peptidase inhibitors (Capnocytophaga ochracea)	WP_026299809.1
RS0102570	I39	family I39 unassigned peptidase inhibitors (Spirosoma linguale)	WP_026299843.1
RS0104815	I39	family I39 unassigned peptidase inhibitors (Chitinophaga pinensis)	WP_026299888.1
RS0106685	I39	family I39 unassigned peptidase inhibitors (Chitinophaga pinensis)	WP_026299924.1
RS0110355	I39	family I39 unassigned peptidase inhibitors (Sulfurospirillum deleyianum)	WP_026299991.1
RS0110975	I39	family I39 unassigned peptidase inhibitor homologues (Bacteroides fragilis)	WP_026300005.1
RS0111085	I39	family I39 unassigned peptidase inhibitors (Chitinophaga pinensis)	WP_026300008.1
RS0112755	I39	family I39 unassigned peptidase inhibitors (Spirosoma linguale)	WP_026300041.1
RS0116215	I39	family I39 unassigned peptidase inhibitors (Chitinophaga pinensis)	WP_026300111.1
RS0116485	I39	family I39 unassigned peptidase inhibitor homologues (Dyadobacter fermentans)	WP_026300120.1
RS0117615	I39	family I39 unassigned peptidase inhibitor homologues (Dyadobacter fermentans)	WP_026300146.1
RS0120355	I39	family I39 unassigned peptidase inhibitors (Spirosoma linguale)	WP_026300208.1
RS0107470	I71	family I71 unassigned peptidase inhibitors (Dictyostelium discoideum)	WP_020212587.1
RS0119030	I87	family I87 unassigned peptidase inhibitors (Treponema vincentii)	WP_020214838.1
RS0107395	I87	family I87 unassigned peptidase inhibitors (Archaeoglobus fulgidus)	WP_026299938.1

Table S4. Carbohydrate active enzymes (CAZymes) in the genome of *F. rivuli* DSM 21788T.

Locus tag	CAZy family	Accession No.
F565_RS0111180	GH1	WP_020213315.1
F565_RS0103420	GH2	WP_020211798.1
F565_RS0106535	GH2	WP_020212402.1
F565_RS0107405	GH2	WP_020212574.1
F565_RS0115755	GH2	WP_020214205.1
F565_RS0115780	GH2	WP_020214210.1
F565_RS0117045	GH2	WP_020214455.1
F565_RS0119430	GH2	WP_020214917.1
F565_RS0120370	GH2	WP_020215100.1
F565_RS0120485	GH2	WP_020215120.1
F565_RS0117575	GH2	WP_026300144.1
F565_RS0117625	GH2	WP_026300147.1
F565_RS0120640	GH2	WP_026300220.1
F565_RS0116205	GH2;CBM32	WP_020214292.1
F565_RS0116190	GH2;CBM57	WP_020214289.1
F565_RS0102030	GH3	WP_020211519.1
F565_RS0109445	GH3	WP_020212978.1
F565_RS0115810	GH3	WP_020214216.1
F565_RS0117205	GH3	WP_020214487.1
F565_RS0118930	GH3	WP_020214819.1
F565_RS0120405	GH3	WP_020215107.1
F565_RS0116480	GH3	WP_026300119.1
F565_RS0120380	GH3	WP_026300211.1
F565_RS0115815	GH3_characterized	WP_020214217.1
F565_RS0120385	GH3_characterized	WP_026300212.1
F565_RS0101095	GH3	WP_020211340.1
F565_RS0102545	GH5	WP_020211622.1
F565_RS0102560	GH5	WP_020211625.1
F565_RS0108150	GH5	WP_020212723.1
F565_RS0101595	GH13	WP_020211437.1
F565_RS0111070	GH13	WP_020213293.1
F565_RS0111110	GH13	WP_020213301.1
F565_RS0111115	GH13	WP_020213302.1
F565_RS0101615	GH130	WP_020211440.1
F565_RS0101090	GH16	WP_020211339.1
F565_RS0101100	GH16	WP_026299811.1
F565_RS0107760	GH23	WP_020212645.1
F565_RS0119115	GH23;CBM50	WP_026300182.1
F565_RS0112370	GH25	WP_020213552.1
F565_RS0102695	GH27	WP_020211653.1
F565_RS0104805	GH27	WP_020212066.1
F565_RS0100465	GH28	WP_020211214.1
F565_RS0100470	GH28	WP_020211215.1
F565_RS0100485	GH28	WP_020211218.1

Continued on next page.

Table S4. (continued)

Locus tag	CAZy family	Accession No.
F565_RS0117255	GH28	WP_020214497.1
F565_RS0100480	GH28;GH105	WP_026299794.1
F565_RS0110815	GH29	WP_020213246.1
F565_RS0120390	GH29	WP_020215104.1
F565_RS0103085	GH30	WP_020211731.1
F565_RS0109450	GH30	WP_020212979.1
F565_RS0120635	GH30	WP_020215150.1
F565_RS0106530	GH31	WP_020212401.1
F565_RS0115750	GH31	WP_020214204.1
F565_RS0120400	GH31	WP_020215106.1
F565_RS0120440	GH31	WP_020215112.1
F565_RS0120375	GH31	WP_026300210.1
F565_RS0116230	GH36_characterized	WP_020214298.1
F565_RS0120890	GH37	WP_026300224.1
F565_RS0106700	GH39	WP_020212436.1
F565_RS0100460	GH42	WP_020211213.1
F565_RS0102555	GH43	WP_020211624.1
F565_RS0103550	GH43	WP_020211823.1
F565_RS0104050	GH43	WP_020211922.1
F565_RS0106520	GH43	WP_020212399.1
F565_RS0111695	GH43	WP_020213417.1
F565_RS0119920	GH43	WP_020215014.1
F565_RS0120410	GH43	WP_020215108.1
F565_RS0120650	GH43	WP_020215152.1
F565_RS0102550	GH43	WP_026299842.1
F565_RS0103540	GH43	WP_026299865.1
F565_RS0106525	GH51	WP_020212400.1
F565_RS0103530	GH51_characterized	WP_020211819.1
F565_RS0103555	GH51_characterized	WP_020211824.1
F565_RS0111105	GH65	WP_020213300.1
F565_RS0112990	GH73;CBM50	WP_020213673.1
F565_RS0116445	GH78	WP_020214337.1
F565_RS0106710	GH88	WP_020212438.1
F565_RS0116200	GH92	WP_020214291.1
F565_RS0118105	GH92	WP_020214658.1
F565_RS0100325	GH95	WP_020211186.1
F565_RS0116195	GH95	WP_020214290.1
F565_RS0117605	GH95	WP_020214566.1
F565_RS0101155	GH97	WP_020211352.1
F565_RS0106465	GH97	WP_020212387.1
F565_RS0103270	GH97	WP_026299855.1
F565_RS0115760	GH97	WP_026300096.1
F565_RS0120575	GH97	WP_026300217.1
F565_RS0100450	GH105	WP_020211211.1
F565_RS0100475	GH105	WP_020211216.1
F565_RS0100440	GH106	WP_020211209.1

Continued on next page.

Table S4. (continued)

Locus tag	CAZy family	Accession No.
F565_RS0116495	GH106	WP_026300121.1
F565_RS0103535	GH127	WP_020211820.1
F565_RS0105955	GH*	WP_020212289.1
F565_RS0115635	GH*	WP_020214182.1
F565_RS0100910	GT2	WP_020211303.1
F565_RS0103365	GT2	WP_020211787.1
F565_RS0104040	GT2	WP_020211920.1
F565_RS0105030	GT2	WP_020212109.1
F565_RS0107490	GT2	WP_020212591.1
F565_RS0107660	GT2	WP_020212624.1
F565_RS0108905	GT2	WP_020212874.1
F565_RS0108925	GT2	WP_020212878.1
F565_RS0111500	GT2	WP_020213379.1
F565_RS0111915	GT2	WP_020213460.1
F565_RS0111920	GT2	WP_020213461.1
F565_RS0112280	GT2	WP_020213533.1
F565_RS0119565	GT2	WP_020214942.1
F565_RS0108240	GT4	WP_020212741.1
F565_RS0108900	GT4	WP_020212873.1
F565_RS0108915	GT4	WP_020212876.1
F565_RS0112235	GT4	WP_020213524.1
F565_RS0112240	GT4	WP_020213525.1
F565_RS0112250	GT4	WP_020213527.1
F565_RS0114870	GT4	WP_020214032.1
F565_RS0119590	GT4	WP_020214947.1
F565_RS0119865	GT4	WP_020215003.1
F565_RS0112260	GT4	WP_026300029.1
F565_RS0109710	GT5	WP_020213029.1
F565_RS0109635	GT9	WP_020213014.1
F565_RS0108930	GT9	WP_026299970.1
F565_RS0119635	GT9	WP_026300194.1
F565_RS0105585	GT19	WP_020212219.1
F565_RS0119305	GT20	WP_026300188.1
F565_RS0113330	GT28	WP_020213740.1
F565_RS0106380	GT30	WP_020212373.1
F565_RS0119215	GT41	WP_020214875.1
F565_RS0110470	GT51	WP_020213177.1
F565_RS0120260	GT51	WP_020215080.1
F565_RS0109675	GT51	WP_026299986.1
F565_RS0110990	GT51	WP_026300007.1
F565_RS0119570	GT*	WP_020214943.1
F565_RS0116970	CBM10;CBM2;GH5	WP_020214441.1
F565_RS0109490	CBM13;GH0	WP_026299981.1
F565_RS0119390	CBM35	WP_020214909.1

Continued on next page.

Table S4. (continued)

Locus tag	CAZy family	Accession No.
F565_RS0113825	CBM35;GH36	WP_020213833.1
F565_RS0105180	CBM50	WP_020212139.1
F565_RS0107955	CBM50	WP_020212684.1
F565_RS0102685	CE2	WP_020211650.1
F565_RS0113710	CE4	WP_020213812.1
F565_RS0102705	CE6	WP_020211655.1
F565_RS0100490	CE7	WP_026299795.1
F565_RS0103195	CE11	WP_020211753.1
F565_RS0100445	CE12	WP_020211210.1
F565_RS0117250	CE12	WP_020214496.1
F565_RS0100415	CE12	WP_026299792.1
F565_RS0100545	CE14	WP_020211231.1
F565_RS0106970	CE14	WP_020212488.1
F565_RS0103010	CE*	WP_020211716.1
F565_RS0116235	CE*	WP_020214299.1
F565_RS0116975	CE*	WP_026300131.1
F565_RS0100420	PL11	WP_026299793.1

* genes attributed to an enzyme class, but not to a family

Table S5. Sulfatases in the genome of *F. rivuli* DSM 21788^T.

Locus tag	Name	Accession No.
F565_RS0105175	Sulfatase	WP_020212138.1
F565_RS0117325	Sulfatase	WP_020214511.1
